# Distraction osteogenesis with temporal bone remodeling for unicoronal craniosynostosis

**DOI:** 10.3171/2021.1.FOCVID20121

**Published:** 2021-04-01

**Authors:** Phuong D. Nguyen, Ahmed Belal, George N. Washington, Matthew R. Greives, David I. Sandberg, Stephen A. Fletcher, Manish N. Shah

**Affiliations:** 1 Division of Plastic Surgery, Department of Surgery; and; 2 Division of Pediatric Neurosurgery, Department of Pediatric Surgery, McGovern Medical School, University of Texas Health Science Center at Houston, Texas

**Keywords:** unicoronal craniosynostosis, distraction osteogenesis, minimally invasive technique

## Abstract

Unicoronal craniosynostosis correction with fronto-orbital advancement and cranial vault remodeling has traditionally been the gold standard. Distraction osteogenesis has the advantage of increased size of movement without constriction of the scalp and decreased morbidity. Although fronto-orbital advancement and cranial vault remodeling are usually performed at 6 months of age or later, distraction osteogenesis is performed at a younger age, between 3 and 6 months, to take advantage of the infant bony physiology. Herein, the authors demonstrate a case of distraction osteogenesis for unicoronal craniosynostosis in a 3-month-old female with significant improvement of her orbital, nasal, and frontal symmetry.

The video can be found here: https://vimeo.com/519047922

## Transcript

**0:26 Patient Presentation**. We present a 3-month-old female with a history of right-sided unicoronal craniosynostosis. Here you see vertical dystopia as well as retrusion of the right forehead. As a sequela of her unicoronal craniosynostosis, she also has nasal root deviation to the affected side as well as temporal bulging on the same side.

**0:44 CT Imaging**. A CT scan is obtained demonstrating the fused right coronal suture and the unicoronal synostosis deformity. A 3D model was created in order to plan the osteotomies as well as to create a vector for optimal distraction.

**0:57 Positioning and Incision**. The patient is positioned on a horseshoe in supine position. A wavy bicoronal incision is created and incised through the galea.

**1:08 Dissection of Scalp and Pericranium**. We then reflect the anterior scalp as well as posterior scalp in a subgaleal fashion. The pericranial flaps are left down as a separate layer. Here we are drawing out specific pericranial flaps based on the lateral superficial temporal vessels as well as the anterior and posterior pedicles. This gives us flexibility in order to raise the flaps as a separate unit, either for camouflage or coverage after the osteotomies.

**1:34 Dissection of Orbits and Nasofrontal Root**. Here we are dissecting out the zygomaticofrontal suture on the right side as well as the right superior lateral orbit. This is done in a subperiosteal dissection using a No. 9 elevator. We are dissecting down as well to the nasal frontal root and the contralateral orbit.

**1:49 Osteotomy Design**. The proposed osteotomies are then marked out. This includes the right fused coronal suture. We are also drawing out a temporal bone wedge. In this patient, the temporal bone is convex. We will ultimately reshape this into a concave position. The placement and vector of the distraction device is drawn out as shown with the arrow to optimize correction of the deformity.

**2:13 Osteotomies—Cranial**. A pterional window is made with a pineapple burr in the right temporal area to create safe access for removal of the fused coronal suture. The location is designed to be just above the greater wing of the sphenoid. The 3D model was critical in planning this location. Here we are creating an osteotomy of the convex right temporal bone using a side-cutting burr. This temporal bone segment will be temporarily removed to create a larger window for visualization and protection of the brain. Previous descriptions of this technique required protection through a suboptimal pterional window.^1–4^

**2:42 Osteotomies—Orbital**. A piezoelectric saw is then used to begin the fronto-orbital osteotomy starting at the zygomaticofrontal suture on the affected side. With direct visualization and protection with malleable retractors, the osteotomy is continued into the orbits. Because calcified structures such as bone do not absorb ultrasonic oscillation, the piezoelectric saw is able to penetrate these structures. Underlying soft tissues vibrate with oscillation and, as such, move away from the vibrating tip. Thus, the dura is protected from the saw. The osteotomy is continued through the medial orbit and across the nasal frontal root. Care is taken to make the nasal osteotomy as low as possible in order to correct the nasal deviation. Once through to the contralateral medial orbit, the osteotomy is continued up until approximately two-thirds of the way to the lateral orbit. A 1-cm segment on the superior orbit at this point is left intact in order to act as a hinge. A perisagittal osteotomy is also made up to the hinge point from the anterior fontanelle.

**3:41 Distractor Placement**. A 30-mm KLS cranial distractor is checked on the back table to ensure that it is functioning properly. A distractor is then inset onto the previously designated area on the temporoparietal bone. The vector is checked such that the distractor will expand in an inferior-medial direction. It is inset with 4-mm screws and care is taken to ensure it is fixated to thick and stable bone, away from any barrel stave segments.

**4:07 Metopic Suture**. If there is instability at the open metopic suture, the two frontal bone segments are buttressed with a spanning resorbable plate. The distractor is expanded in situ to confirm adequate vector and correction of the unicoronal deformity. Once the distractor placement is confirmed, it is reset into the resting position. A separate stab incision is made in the posterior scalp to accommodate the turning arm.

**4:28 Temporal Bone Remodeling and Closure**. The temporalis is resuspended, and the scalp will be closed in two layers. Prior to closure, the temporal bone wedge is replaced such that it is inside out and now concave. It is fixated with a spanning resorbable plate.

**4:39 Postoperative Imaging**. A postoperative AP and lateral x-ray demonstrate starting position prior to activation phase.

**4:44 Early Postoperative Photos**. Here we see this patient at only 2 weeks postoperative in the middle of activation. There is already improvement of the orbital symmetry and frontal bone position.

**4:50 Second Case Example With 1-Year Follow-Up**. Here are preoperative and 1-year postoperative images in a patient who underwent the same procedure. The orbital, nasal, and forehead symmetry are significantly improved.

**4:59 Second Case CT**. Pre- and postoperative CT scans demonstrate improved bony symmetry in both orbits as well as moving the nasal root to midline and correction of the frontal retrusion.

**5:07 Conclusion**. In conclusion, distraction osteogenesis for unicoronal synostosis is an effective technique with decreased morbidity that should be considered.

## Author Contributions

Primary surgeon: Nguyen, Greives, Sandberg, Fletcher, Shah. Assistant surgeon: Belal. Editing and drafting the video and abstract: Nguyen, Belal, Washington, Shah. Critically revising the work: Nguyen, Washington, Greives, Shah. Reviewed submitted version of the work: Nguyen, Belal, Washington, Greives, Shah. Approved the final version of the work on behalf of all authors: Nguyen. Supervision: Nguyen.
